# Case report: pseudoaneurysm of left ventricle secondary to infective endocarditis complicated by cardiac rupture—a multimodality imaging approach

**DOI:** 10.1093/ehjcr/ytae318

**Published:** 2024-07-02

**Authors:** Sara Ruggerini, Andrea Venturelli, Alberto Giovanni Tripodi, Carlotta Brega

**Affiliations:** Cardiology Unit, Ospedale ‘Ceccarini’, Riccione (Rimini), Azienda USL della Romagna, Viale Frosinone, 7, Riccione, RN 47838, Italy; Cardiology Unit, Ospedale ‘Ceccarini’, Riccione (Rimini), Azienda USL della Romagna, Viale Frosinone, 7, Riccione, RN 47838, Italy; Cardiovascular Department, Maria Cecilia Hospital, GVM Care & Research, Cotignola, Ravenna, Italy; Cardiovascular Department, Maria Cecilia Hospital, GVM Care & Research, Cotignola, Ravenna, Italy

**Keywords:** Case report, Pseudoaneurysm, Endocarditis, Multimodality imaging approach

## Abstract

**Background:**

Pseudoaneurysm (PSA) of the left ventricle (LV) is a rare peri-annular complication of infective endocarditis (IE), and it is associated with high risk of free wall rupture. The diagnosis is challenging because the exact incidence and the pathogenesis are still unclear.

**Case summary:**

A 69-year-old lady underwent prosthetic mitral valve replacement for IE secondary to *Staphylococcus aureus* sepsis complicated by multiple embolizations. In the post-operative period, the patient developed persistent low-grade fever with negative blood culture. Transoesophageal echocardiography (TOE) revealed complete posterior valve detachment and a PSA sac arising from the antero-lateral commissure; the colour flow Doppler showed massive mitral regurgitation. Thoracic computed tomography (CT) scan confirmed the echo data and the exact localization of the cardiac rupture. The patient underwent reoperation, a pericardial patch was sutured to exclude the PSA sac, and a mechanical prosthesis valve was finally implanted. A follow-up TOE revealed the exclusion of the PSA; two leakages with mild peri-valvular mitral regurgitation were found, with no haemodynamic impact.

**Discussion:**

In our case, the patient developed a PSA of the LV as a consequence of peri-annular extension of IE on the mitral valve. Pseudoaneurysm is a potentially lethal complication, if not promptly treated. Multimodality imaging including echocardiography and CT scan is recommended, in order to plan surgery *ad hoc*.

**Learning points**

- Cardiac rupture secondary to left ventricle pseudoaneurysm is a rare and potentially fatal complication of prosthetic valve infection.
- Echocardiography still plays a central role in the timely diagnosis but a multimodality imaging approach is recommended to plan prompt surgical strategy.
- The preferable approach for infective endocarditis with peri-annular involvement is still a matter of debate although surgical treatment seems to be the strategy of choice with good outcome despite the high-risk surgery.

## Introduction

Pseudoaneurysm (PSA) of the left ventricle (LV) is a rare peri-annular complication of infective endocarditis (IE) associated to high risk of free wall rupture. The diagnosis is challenging, and the exact incidence and the pathogenesis are still unclear. The mortality rate is high due to the risk of spontaneous rupture (30–45%), especially in the absence of surgical treatment.^1^

A multimodality imaging approach including echocardiography and angio-computed tomography (CT) scan is recommended, in order to plan *ad hoc* surgery.

We report a case of PSA of the LV as a consequence of peri-annular extension of IE on the mitral valve, complicated by cardiac rupture.

## Summary figure

**Figure:**
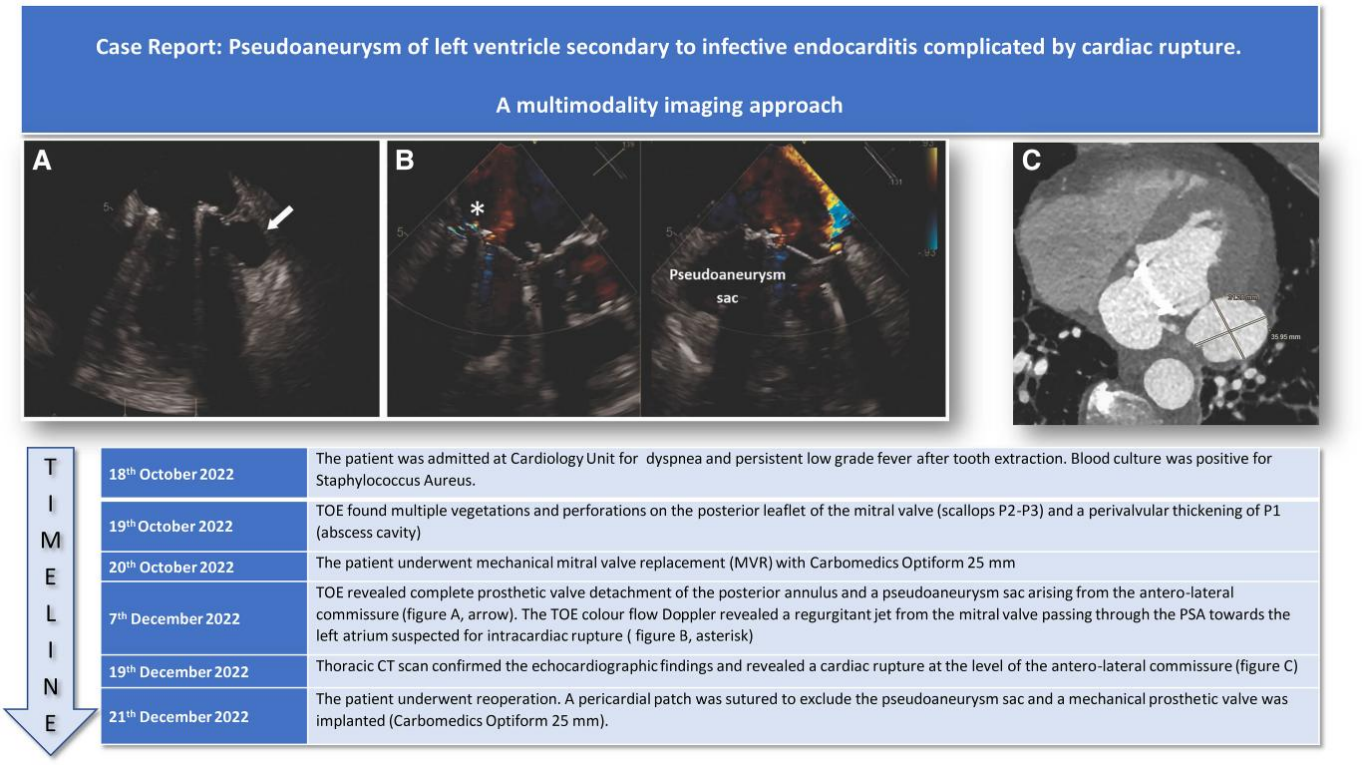


## Case presentation

A 69-year-old woman was admitted to the cardiology unit for evaluation of dyspnoea and persistent low-grade fever after tooth extraction. Physical examination revealed a pansystolic murmur at the mitral area in addition to multiple splinter haemorrhages.

**Figure 1:**
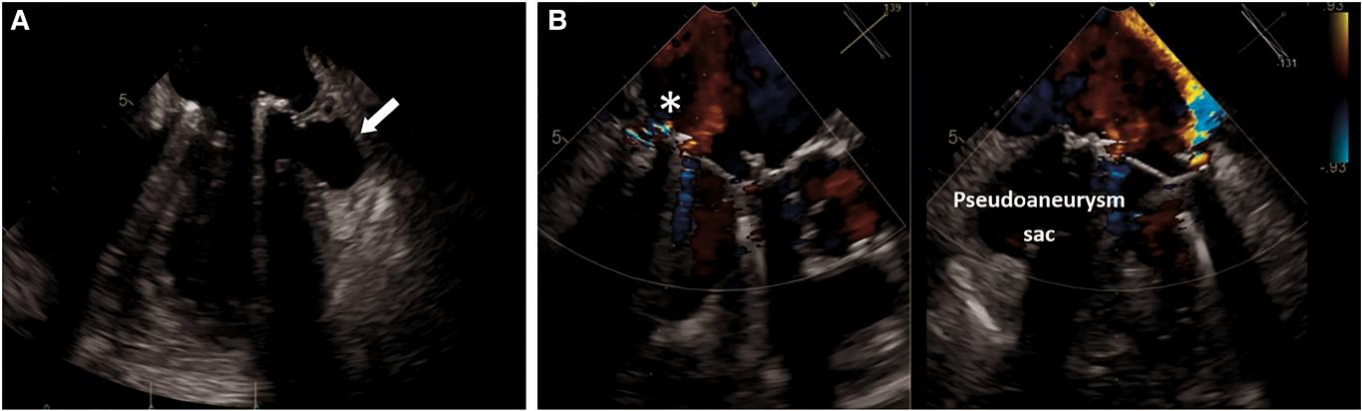
(*A*) Mid-oesophageal four-chamber view showing pseudoaneurysm arising from the basal postero-lateral wall of the left ventricle. (*B*) X-plane mid-oesophageal long-axis view: colour Doppler shows the flow through the cavity between the left atrium and left ventricle (arrows).

**Figure 2:**
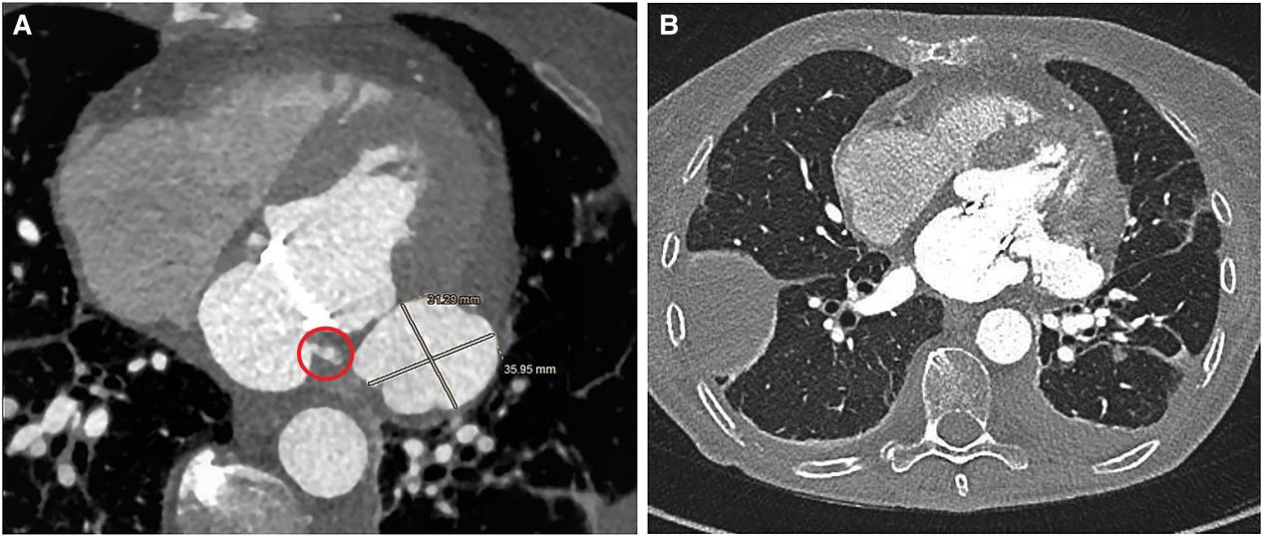
(*A*) and (*B*) Axial cardiac computed tomography angiography demonstrating the left ventricular pseudoaneurysm at the level of antero-lateral commissure.

Her past medical history was significant for Sjogren syndrome, and she was under steroid therapy. Blood cultures were positive to methicillin-susceptible *Staphylococcus aureus* (MSSA), and a CT scan revealed multiple cerebral and renal embolizations. The transoesophageal echocardiography showed multiple vegetations and perforations on the posterior leaflet of the mitral valve (P1–P2 scallop), a peri-valvular abscessual cavity of the posterior annulus in P1 zone and significant mitral regurgitation (see Supplementary material online, *Video S1*). The patient was treated with antimicrobial therapy using oxacillin and meropenem at first time and then switched to cefazolin based on the antibiogram results. The patient’s clinical course was complicated by pulmonary oedema that required intravenous therapy with diuretics and inotropes. A mechanical mitral valve (Carbomedics Optiform 25 mm) was implanted. Cultures of native mitral valve were positive for MSSA. The antibiotic regime was given for 6 week after the operation. After a few weeks, the patient was affected by persistent low-grade fever despite negative blood cultures. Transoesophageal echocardiography revealed a complete posterior valve detachment (see Supplementary material online, *Video S2*) and a PSA originating from the antero-lateral commissure of the mitral ring (*Figure 1A* and *B*). To complete the screening, an angio-CT scan was performed, which confirmed the diagnosis and identified the exact localization of the cardiac rupture on the antero-lateral commissure (*Figure 2A* and *B*). The patient underwent reoperation through complete median sternotomy. Cardiopulmonary bypass was established through ascending aorta and both superior and inferior caval veins, and cardiac arrest was obtained using antegrade cold blood cardioplegia. Through left atriotomy, access to the mitral valve was gained. Once exposed, a complete posterior intercommissural periprosthetic leakage was observed. After removing the valve, a 3.5 cm tear in the ventricular wall at the level of the antero-lateral commissure was detected. A pericardial patch was sutured in order to repair the tear and to exclude the PSA sac: single-pledgeted stiches were passed from the ventricular side towards the atrial side, through the mitral annulus. As a reinforcement, another pericardial band was sutured above the annulus. At the end, a mechanical prosthesis was implanted. Cultures of mitral valve prosthesis were positive to *Staphylococcus warneri*. Although intraoperative finding raised some concerns about possible IE on prosthetic mitral valve, oxacillin 12 g/day was reintroduced for 6 weeks.

Post-operative echocardiography revealed the complete exclusion of the PSA and absence of para-valvular leaks. A follow-up TOE confirmed the exclusion of the PSA and showed two small leaks (the major at the level of the antero-lateral commissure) with mild peri-valvular mitral regurgitation without haemodynamic impact (see Supplementary material online, *Video S3*).

## Discussion

Left ventricle PSA is a rare complication of mitral IE that occurs in about 1% of cases after MVR.^1^ Pseudoaneurysm is the result of rupture of the ventricular free wall, contained by the pericardium or surrounding tissue. When this occurs, the mortality rate is high due to the risk of spontaneous rupture (30–45%), especially if not treated with surgery.^1^ More often, intrapericardial rupture occurs resulting in cardiac tamponade and death, while contained rupture evolving in PSA is rare. Pseudoaneurysms are usually located posteriorly, as described in our case. The exact pathogenesis of peri-annular PSA endocarditis is still unclear, and there are two hypotheses that are most pursued. The widely supported theory suggests that the bacterial invasion of the mitral ring causes necrosis and weakening of the cardiac wall, resulting in the formation of an abscess cavity with a fistula to the ventricle, which progresses to PSA. According to the second theory, an eroded myocardial area can expand gradually under the high LV pressure, leading to a dissection of the portion of the LV and eventually the PSA.^2,3^ In our case, the second hypothesis seems to be the most likely, since the trigger event (dental surgery) had occurred several weeks ago. The diagnosis of PSA is challenging and requires a prompt work-up for the risk of rupture.

Differential diagnosis includes true aneurysm of LV, which differs because of the presence of thinned dyskinetic myocardium region that involves the full thickness of the wall, and valve ring abscess, from which it differs due to the presence of a communication with the left cardiac chambers. In our case, the suspicion arose from TOE.

Transoesophageal echocardiography still plays a central role and provides a clear view of the posterior structure of the heart although it is not sufficient, with a need for more specific imaging techniques to identify PSA. In this case, the TOE colour flow Doppler revealed a regurgitant jet from the mitral valve passing through the PSA towards the left atrium suspected for intracardiac rupture at the level of the antero-lateral commissure; CT scan has been crucial in order to locate the exact position of cardiac breach. Computed tomography scan has a higher sensitivity compared with TOE for detection of abscess and PSA (78 vs. 69%) reaching 87% when the findings were restricted to multiphase CT studies.^4^

The appropriate approach to IE with peri-annular involvement is still a matter of debate. To date, no randomized trials have been conducted, and both medical strategy alone and combined medical/surgical strategy are accepted.^5^ A surgical approach should be required to prevent the spontaneous rupture of the PSA and to eradicate the infection. In our case, surgical treatment required mitral valve replacement, wide resection of the abscess cavity, and patch closure of PSA with good outcome and prognosis.^1^ The use of intra-cavitary patch is considered the treatment of choice, because it preserves the geometry of the mitral valve ring.^6^ In our patient, a patch of bovine pericardium was sutured to close the PSA communication with the LV, and a mechanical prosthetic mitral valve was implanted. A 3-month follow-up TOE revealed closure of PSA, normal function of the prosthetic mitral valve, and presence of two small leaks with no haemodynamic impact. Although current evidence does not give a clear surgical timing, our case confirms that surgery is the appropriate treatment for PSA to eradicate the infection and correct haemodynamic distress.^7–9^ Surgery for PSA is at high risk, with higher post-operative mortality and morbidity rates (3.7–31%) than in those with uncomplicated active endocarditis.^10–12^ In our patient, the risk was especially high, considering the re-operation; the post-operative course was uneventful suggesting that the expertise of the surgeon is certainly pivotal.

## Conclusion

Pseudoaneurysm is a rare and potentially lethal complication of IE. The diagnosis of PSA is challenging for the imaging, that is why a multimodality approach should be adopted. Transoesophageal echocardiography still plays a central role in the diagnosis, but due to its higher sensitivity, cardiac angio-CT scan has a complementary role in the diagnostic work-up and surgical planning. Although the treatment of choice is still a matter of debate, surgical treatment is recommended to avoid the risk of rupture, despite the high-risk surgery, especially for the re-intervention.

## Lead author biography


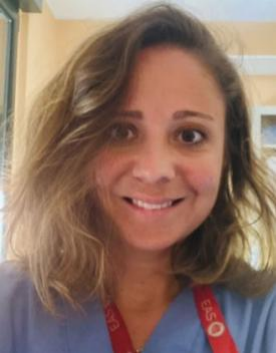


Sara Ruggerini is consultant cardiologist at Ospedale Ceccarini in Riccione. Her field of interest is valvular heart diseases; she mainly works in the echocardiographic laboratory. She is a member of several scientific societies as Italian Society of Cardiology (SIC) and European Society of Cardiology (ESC).

## Supplementary Material

ytae318_Supplementary_Data

## Data Availability

The data underlying this article cannot be shared publicly due to the privacy of individuals that participated in the study. The data will be shared on reasonable request to the corresponding author.

## Supplementary material

**Consent:** The authors confirm that written consent for submission and publication of this case report including images and associated text has been obtained from the patient in line with the COPE guidelines.

**Funding:** None declared.

## References

[1] Frances C, Romero A, Grady D. Left ventricular pseudoaneurysm. J Am Coll Cardiol 1998;32:557–561. 9741493 10.1016/s0735-1097(98)00290-3

[2] Graupner C, Vilacosta I, SanRomán JA, Ronderos R, Sarriá C, Fernández C, et al Periannular extension of infective endocarditis. J Am Coll Cardiol 2002;39:1204–1211. 11923047 10.1016/s0735-1097(02)01747-3

[3] Tingleff J, Egeblad H, Gøtzsche C-O, Baandrup U, Kristensen BØ, Pilegaard H, et al Perivalvular cavities in endocarditis: abscesses versus pseudoaneurysms? A transesophageal Doppler echocardiographic study in 118 patients with endocarditis. Am Heart J 1995;130:93–100. 7611130 10.1016/0002-8703(95)90241-4

[4] Oliveira M, Guittet L, Hamon M, Hamon M. Comparative value of cardiac CT and transesophageal echocardiography in infective endocarditis: a systematic review and meta-analysis. Radiol Cardiothorac Imaging 2020;2:e190189. 33778583 10.1148/ryct.2020190189 PMC7977865

[5] San Román JA, Vilacosta I, Sarriá C, de la Fuente L, Sanz O, Vega JL, et al Clinical course, microbiologic profile, and diagnosis of periannular complications in prosthetic valve endocarditis. Am J Cardiol 1999;83:1075–1079. 10190523 10.1016/s0002-9149(99)00018-1

[6] Esposito F, Renzulli A, Festa M, Cerasuolo F, Caruso A, Sarnicola P, et al Submitral left ventricular aneurysm. Report of 2 surgical cases. Texas Heart Inst J 1996;23:51–53. PMC325303 8680275

[7] Gueron M, Hirsch M, Venderman K, Freund H, Borman J. Pseudoaneurysm of left ventricle. Report of a case diagnosed by angiography and successfully repaired. Br Heart J 1973;35:663–665. 4712473 10.1136/hrt.35.6.663 PMC458677

[8] Smith RC, Goldberg H, Bailey CP. Pseudoaneurysm of the left ventricle: diagnosis by direct cardioangiography; report of two cases successfully repaired. Surgery 1957;42:496–510. 13467606

[9] Yakierevitch V, Vidne B, Melamed R, Levy MJ. False aneurysm of the left ventricle. Surgical treatment. J Thorac Cardiovasc Surg 1978;76:556–558. 703362

[10] David TE, Regesta T, Gavra G, Armstrong S, Maganti MD. Surgical treatment of paravalvular abscess: long-term results. Eur J Cardiothorac Surg 2007;31:43–48. 17140802 10.1016/j.ejcts.2006.10.036

[11] Naqvi TZ, Boyatt J, Siegel RJ. Predictors of mortality in paravalvular abscess. J Am Soc Echocardiogr 2005;18:1404–1408. 16376774 10.1016/j.echo.2005.06.007

[12] Knosalla C, Weng Y, Yankah AC, Siniawski H, Hofmeister J, Hammerschmidt R, et al Surgical treatment of active infective aortic valve endocarditis with associated periannular abscess–11 year results. Eur Heart J 2000;21:490–497. 10681490 10.1053/euhj.1999.1877

